# Epidemiology of Lyme Borreliosis in France in Primary Care and Hospital Settings, 2010–2019

**DOI:** 10.1089/vbz.2022.0050

**Published:** 2023-04-12

**Authors:** Charles Nuttens, Antoine Bessou, Stéphanie Duret, Jozica Skufca, Emmanuelle Blanc, Andreas Pilz, Bradford D. Gessner, Jean-François Faucher, James H. Stark

**Affiliations:** ^1^ Vaccine Medical Development, Scientific and Clinical Affairs, Pfizer SAS, Paris, France.; ^2^ IQVIA Opérations France, Courbevoie, France.; ^3^ Pharmacovigilance and Epidemiology Services, P95, Leuven, Belgium.; ^4^ Vaccine Medical Development, Scientific and Clinical Affairs, Pfizer Corporation Austria, Vienna, Austria.; ^5^ Vaccine Medical Development, Scientific and Clinical Affairs, Pfizer, Inc., Collegeville, Pennsylvania, USA.; ^6^ Service des maladies infectieuses et tropicales, CHU de Limoges, Limoges, France.

**Keywords:** Lyme borreliosis, Lyme disease, tick-borne disease, epidemiology, erythema migrans, France

## Abstract

**Introduction:**

Lyme borreliosis (LB) is a growing public health concern requiring accurate and comprehensive epidemiological knowledge to inform health care interventions. This study compared the epidemiology of LB in primary care and hospital settings, using for the first time in France three sources of data, and highlighted specific populations at higher risk of developing LB.

**Methods:**

This study analyzed data from general practitioner networks (*i.e*., Sentinel network, Electronic Medical Records [EMR]) and the national hospital discharge database to describe the LB epidemiology from 2010 to 2019.

**Results:**

The average annual incidence rates of LB in primary care increased from 42.3 cases/100,000 population in 2010–2012 to 83.0/100,000 in 2017–2019 for the Sentinel Network and 42.7/100,000 to 74.6/100,000 for the EMR, following a marked rise in 2016. The annual hospitalization rate remained stable from 2012 to 2019 fluctuating between 1.6 and 1.8 hospitalizations/100,000. Women were more likely to present with LB in primary care setting compared with men (male-to-female incidence rate ratio [IRR] = 0.92), whereas men were predominant among hospitalizations (IRR = 1.4), with the largest discordance among adolescents aged 10–14 years (IRR = 1.8) and adults aged 80 years and older (IRR = 2.5). In 2017–2019, the average annual incidence rate peaked among persons aged 60–69 years in primary care (>125/100,000) and aged 70–79 years among hospitalized patients (3.4/100,000). A second peak occurred in children aged 0–4 or 5–9 years depending on sources. Incidence rates in Limousin and the north-eastern regions were the highest for both primary care and hospital settings.

**Conclusions:**

Analyses showed disparities in the evolution of incidence, sex-specific incidence rates, and predominant age groups between primary care and hospital settings that merit further exploration.

## Introduction

Lyme borreliosis (LB) is a bacterial infection caused by *Borrelia burgdorferi sensu* lato, a group of diverse borrelia genospecies including *B. burgdorferi* stricto *sensu*, *Borrelia afzelii* and *Borrelia garinii*, transmitted by the bite of an infected *Ixodes* genus tick (Rudenko et al, 2011). Erythema migrans (EM) is the most common clinical manifestation, usually seen in the primary care setting (Kullberg et al, 2020). More severe manifestations such as Lyme neuroborreliosis, Lyme arthritis, Lyme carditis, and acrodermatitis chronica atrophicans can develop within weeks to years following dissemination of the pathogen and may require hospitalization (Kullberg et al, 2020; Moniuszko-Malinowska et al, 2018).

Evaluation of the epidemiology of LB is critical to inform the population of the risk, determine the most appropriate health care intervention, and evaluate their impact. A distinct analysis of the incidence rates in primary care and hospital settings is essential to accurately capture the overall burden of LB and the interrelatedness. For example, an increase in the number of general practitioner (GP) consultations for LB in primary care, associated with appropriate treatment, could lead to a reduction of disseminated LB cases and thereby hospitalizations.

In France, LB hospitalizations are extracted from the national hospital discharge database (*i.e*., Programme de Médicalisation des Systèmes d'Information [PMSI]) using Lyme-specific *International Classification of Diseases, 10th Revision* (ICD-10) codes. This national database includes the hospitalization records of the entire French population that capture the complete reported number of hospitalizations for LB. A detailed analysis of the data through 2016 has been published (Septfons et al, 2019) and the annual incidence rates are communicated annually (Santé Publique France, 2022). The annual LB hospitalization rate fluctuated between 1.1 and 1.5 hospitalization per 100,000 population between 2005 and 2019 (Santé Publique France, 2022). Important regional disparities were observed, with Limousin and Alsace being the two regions with the highest hospitalization rates (>4/100,000) (Septfons et al, 2019).

LB epidemiology in primary care is derived from the national Sentinel network (*i.e*., Réseau Sentinelles), a surveillance system that collects, processes, and publishes LB epidemiological data from a network of volunteer GPs since 2009 (Flahault et al, 2006). The majority of cases reported by the Sentinel GPs were patients with EM and only a few had a disseminated form of the disease (Sentinelles, 2019). In 2019, the national incidence rate was 76/100,000 (95% confidence interval [CI], 65–87/100,000), representing 50,133 cases (95% CI, 43,029–57,237) (Sentinelles, 2019). Limousin is regularly the region with the highest incidence rate in primary care, exceeding 500/100,000 in 2015 and 2016, followed by the north-eastern regions (*i.e*., Alsace, Lorraine) with incidence rates frequently around 250/100,000 (Sentinelles, 2019).

Another GP network exists in France but has never been used to investigate the epidemiology of LB. The Electronic Medical Records (EMR) from a GP network independent of the Sentinel network have been frequently used to assess or describe the management of various diseases in primary care (Joumaa et al, 2022; Maravic et al, 2018). The organization of this network is different compared with the Sentinel network. Sentinel GPs actively report cases including disease details that allow a group of experts to validate the LB cases. One limitation of this approach is the restricted participation, with ∼350–550 active GPs nationwide, which might impact the accuracy of the incidence rate estimation when performing analysis by subgroup. In contrast, the GPs participating in the EMR network record in a specific software the diagnosis during the consultation with their patients, which are coded for further analyses. Approximately 1200 GPs participate in the generation of the EMR database nationwide, which allows more accurate estimation by sex and age group.

Although the epidemiology of LB has been well characterized in France, the most recent comparison between primary care and hospital epidemiology studied data through 2016. This study analyzed and compared LB incidence data in primary care setting from 2010 to 2019 and hospital setting from 2012 to 2019 to characterize the epidemiology of LB in France and identify the most recent trends. We analyzed for the first time LB data from EMR of a network of GPs independent of the Sentinel network, to assess the incidence of LB in primary care setting complementary to the Sentinel network estimates.

## Methods

### Data sources

LB surveillance data were extracted from three independent sources. Primary care data were derived from the French Sentinel network and the EMR database. Hospital data were extracted from the PMSI.

The French Sentinel network is a real-time epidemiologic surveillance system composed of ∼350–550 active GPs located throughout mainland France, representing 0.5–0.9% of the French GP population. Since 2009, they reported newly diagnosed LB every week, which were validated by a group of experts including clinicians, microbiologists, and epidemiologists using the European Union Concerted Action on Lyme Borreliosis (EUCALB) case definitions (Stanek et al, 2011). Thereafter, data were extrapolated to estimate the total number of cases and incidence rates at national level, by region and age.

The EMR dataset was generated through extraction of anonymized, patient-level information from ∼1200 active GPs, representing ∼2% of all the French GP population. GPs from this network most likely do not participate in the Sentinel network. The panel of GPs is maintained to ensure representative of the GP practice population in terms of age, sex, type of practice (*e.g*., partial, private activity), and geographical area of activity (Joumaa et al, 2022; Maravic et al, 2018). Data were collected in real-time during consultations via a dedicated medical software. LB cases were derived from GP diagnoses identified using a textual database search, and were therefore not validated by a group of experts. For patients with more than one GP visit with a diagnosis of LB within the study period, only visits separated by 12 months or more from the previous visit were considered. Data were extrapolated to estimate the total number of cases and incidence rates at a national level, by region, by age, by sex, and by age and sex.

PMSI is the French national hospital discharge database collecting information after each hospital stay, for the entire population in France. A unique patient identifier allows the identification of multiple hospitals stays for the same patient. A hospital discharge record was produced for each inpatient stay. The record included the reason of hospitalization as the primary diagnosis (PD), related medical conditions as the associated diagnoses (AD), and the patients' characteristics (age, sex, place of hospitalization, and residence). PD and AD were coded with ICD-10.

LB cases hospitalized between January 1, 2012 and December 31, 2019 in mainland France were identified through the following criteria: (1) LB diagnosis recorded as PD using the ICD-10 code A69.2 (Lyme disease), M01.2 (arthritis in Lyme disease) or L90.4 (Acrodermatitis chronica atrophicans), or (2) LB diagnosis recorded as AD and ICD-10 codes compatible with LB symptoms recorded as the PD (neurological, cardiac, articular, and ocular disorders). LB symptoms and their ICD-10 codes that were used are defined elsewhere (Septfons et al, 2019). Hospital discharge reports with no patient identifier were excluded. For patients with more than one hospitalization with a diagnosis of LB within the study period, only hospitalizations separated by 12 months or more from the previous visit were considered.

This article is based on data from GP networks and the national hospital discharge database and does not contain any new studies with human participants or animals performed by any of the authors; therefore, Institutional Review Board approval was not required.

### Statistical analyses

#### Incidence rates in primary care

Data from 2010 to 2019, the 10 most recent years for which data were available at the initiation of the study, were analyzed for the primary care sources. For Sentinel network data, incidence rates and their 95% CIs were provided by the Sentinel network. Incidence rates from the EMR were estimated using an extrapolation process similar to the Sentinel network: for a geographical area in mainland France, the average number of LB cases reported per GP in the area was multiplied by the total number of GP practicing in the area divided by the population of the area (estimated by the National Institute of Statistics and Economic Studies). CIs were estimated assuming the number of reported cases followed a Poisson distribution. National incidence rates were then estimated by adding all incidence rates calculated at the regional levels.

#### Hospitalization rates

LB cases hospitalized over the 2012–2019 period, all the years for which patient-level data were available at the initiation of the study, were analyzed. For each region, the hospitalization rate was directly obtained by dividing the number of hospital stays in the region by the population of the region. Extrapolation and CI calculation were unnecessary as PMSI database integrates all hospitalizations in France and is considered complete. The place of residence of the hospitalized patients was used to determine the regional incidence rates.

For all data sources, incidence rates were calculated by age group and region. In addition, incidence rates were calculated for sex and sex and age from the PMSI and the EMR. The Sentinel network did not publish extrapolated sex-specific data.

#### Average annual incidence rate

The average annual incidence rates for 2010–2012 and 2017–2019 were calculated to smooth the annual variability and to demonstrate the change over the study period. The most recent 3-year period (2017–2019) was used to describe the regional and age-specific incidence rates. Ninety-five percent CIs were calculated over the 3-year periods using the method of variance estimates recovery (MOVER) approach (Donner and Zou, 2012).

#### Male-to-female incidence rate ratio

Male-to-female incidence rate ratio (IRR) was calculated by dividing the incidence rate estimated in men to the incidence rate estimated in women for the period studied or age group. Ninety-five percent CIs were calculated using the MOVER approach (Donner and Zou, 2012).

SAS software version 9.4 (SAS Institute, Cary, NC) was used for incidence rate estimations, and R 3.5.0 (R Foundation for Statistical Computing, Vienna, Austria) was used to perform the statistical analyses and generate the figures.

## Results

### Evolution of LB incidence and hospitalization rates

**FIG. 1.**
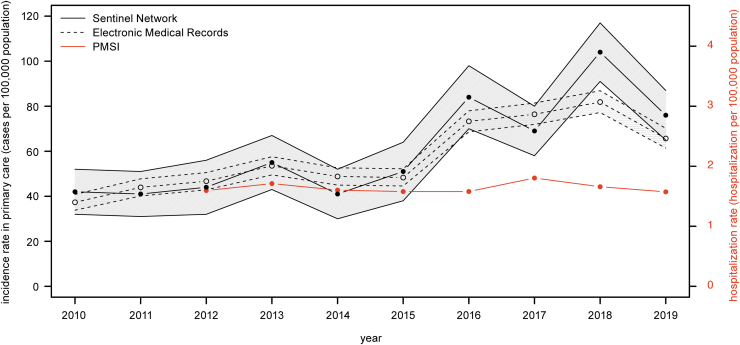
Evolution of incidence rates of LB in primary care and hospital settings in France, 2010–2019. Primary care incidence rates are extrapolated from the Sentinel network (*black filled circle*) and the EMR (*gray empty circle*) and hospitalization rates from the PMSI (*red filled circle*). The lower and upper confidence intervals are provided for the Sentinel network and the EMR. *Left Y*-*axis* represents the incidence rate in primary care and the *right Y*-*axis* represents the hospitalization rate. EMR, Electronic Medical Records; LB, Lyme borreliosis; PMSI, Programme de Médicalisation des Systèmes d'Information (French national hospital discharge database).

The average annual incidence rate of LB increased from 42.3/100,000 (95% CI, 36.2–48.5/100,000) in 2010–2012 to 83.0/100,000 (95% CI, 76.2–89.8/100,000) in 2017–2019 according to the Sentinel network estimates, representing an increase of 96.1% (Table 1). The trend was similar for the average annual incidence rate of LB using the EMR, with an increase from 42.7/100,000 (95% CI, 40.5–44.8/100,000) in 2010–2012 to 74.6/100,000 (95% CI, 72.1–77.4/100,000) in 2017–2019, representing an increase of 75.0%. The incidence rate was stable from 2010 to 2015, followed by a marked increase observed in 2016 for both surveillance systems (Fig. 1). In comparison, the LB hospitalization rate was stable over the study period fluctuating between 1.57 and 1.80 hospitalizations/100,000 (Fig. 1). The average annual hospitalization rate was 1.7/100,000 in 2017–2019 (Table 1).

**Table 1.** Average Incidence Rates (per 100,000 Population), Number of Cases, and Male-To-Female Incidence Rate Ratio of Lyme Borreliosis in Primary Care (*i.e*., Sentinel Network, Electronic Medical Records) and Hospital (*i.e.,* Programme de Médicalisation des Systèmes d'Information) Settings in France, 2010–2012 and 2017–2019

		Sentinel network	EMR	PMSI
		Estimate (95% CI)	Estimate (95% CI)	
Average annual incidence rate				
2010–2012	/100,000	42.3 (36.2–48.5)	42.7 (40.5–44.8)	1.6^a^
2017–2019	/100,000	83.0 (76.2–89.8)	74.6 (72.1–77.4)	1.7
Relative increase^b^	%	96.1	75.0	5.2
Average annual number of cases				
2010–2012	*n*	26,737 (22,740–30,733)	26,911 (25,557–28,265)	1011^a^
2017–2019	*n*	54,447 (49,998–58,897)	48,434 (46,693–50,175)	1087
Male-to-female incidence rate ratio				
2010–2012		NA	0.88 (0.80–0.96)	1.44^a^
2017–2019		NA	0.91 (0.85–0.98)	1.46
Age-specific average annual incidence rate, 2017–2019				
0–4 Years	/100,000	65.3 (39.5–91.1)	24.9 (18.5–31.3)	0.53
5–9 Years	/100,000	51.7 (30–73.4)	45.8 (37.3–54.2)	2.03
10–14 Years	/100,000	35.3 (18.5–52.2)	31.8 (24.7–38.9)	1.06
15–19 Years	/100,000	33.7 (17.1–50.7)	30.9 (23.9–37.9)	0.71
20–29 Years	/100,000	49.3 (33.8–64.9)	51.2 (44.6–57.9)	0.72
30–39 Years	/100,000	64.7 (47.6–81.7)	68.7 (61.4–75.9)	1.19
40–49 Years	/100,000	93.7 (73.8–113.6)	74.7 (67.3–82.1)	1.61
50–59 Years	/100,000	117.7 (95.5–139.9)	112.2 (103.1–121.2)	2.16
60–69 Years	/100,000	156.3 (128.9–183.7)	127.3 (117.1–137.4)	2.79
70–79 Years	/100,000	124.0 (94.4–153.6)	123.4 (111–135.7)	3.35
80+ Years	/100,000	47.7 (26.6–68.7)	48.0 (39.3–56.6)	1.58
Subnational average annual incidence rate, 2017–2019				
Alsace	/100,000	244.3 (180.2–308.5)	162.3 (138.2–186.4)	3.35
Aquitaine	/100,000	44.7 (17.9–71.9)	88.0 (74.8–101.1)	1.96
Auvergne	/100,000	114.7 (81–148.3)	153.3 (127.6–179.3)	2.57
Basse-Normandie	/100,000	15.7 (6.1–30.6)	23.3 (9.9–38.2)	1.38
Bourgogne	/100,000	83.7 (41.2–128.5)	97.0 (79.4–114.2)	2.35
Bretagne	/100,000	54.3 (30.9–78.1)	56.3 (44.6–68.1)	1.9
Centre	/100,000	61.3 (43–79.7)	53.7 (42.4–64.9)	2.68
Champagne-Ardenne	/100,000	84.7 (50.2–119.8)	115.3 (96.3–134.6)	2.98
Corse	/100,000	24.3 (10–40.5)	60.3 (30.4–91.1)	0.2
Franche-Comté	/100,000	110.3 (67–153.7)	116.3 (88.7–144.1)	3.17
Haute-Normandie	/100,000	45.3 (17.6–80)	30.3 (21.3–39.7)	1.33
Ile-de-France	/100,000	37.7 (25.7–49.6)	52.7 (47.5–57.8)	1.13
Languedoc-Roussillon	/100,000	27.0 (7.4–47.2)	47.3 (35.5–59.7)	0.97
Limousin	/100,000	330.0 (185.7–474.3)	355.3 (299–411.7)	8.15
Lorraine	/100,000	207.7 (149.7–265.7)	110.0 (92.1–128.2)	2.95
Midi-Pyrénées	/100,000	83.0 (51.6–114.4)	93.7 (80.6–106.7)	1.25
Nord-Pas-de-Calais	/100,000	35.3 (15.9–55.3)	28.3 (21.9–34.7)	0.94
Pays-de-la-Loire	/100,000	59.0 (34.8–83.2)	46.0 (38.5–53.4)	0.81
Picardie	/100,000	37.7 (14.1–63)	62.3 (48.7–75.9)	2.04
Poitou-Charentes	/100,000	60.7 (15.8–124.8)	66.0 (51.2–80.7)	1.96
Provence-Alpes-Côte d'Azur	/100,000	66.3 (41–91.6)	58.7 (50.7–66.3)	0.39
Rhône-Alpes	/100,000	206.3 (178.7–234)	111.3 (101.3–121.8)	2.02

^a^ Data for 2012 only.

^b^ Evolution of average incidence rate between 2010 and 2012 (Sentinel network, EMR) or 2012 (PMSI) and 2017–2019, expressed in relative difference.

CI, confidence interval; EMR, Electronic Medical Records; NA, not available; PMSI, Programme de Médicalisation des Systèmes d'Information (French national hospital discharge database).

These incidence rates represented 54,447 (95% CI, 49,998–58,897) cases annually, averaged over the 2017–2019 period, according to the Sentinel network, 48,434 (95% CI, 46,693–50,175) cases using the EMR data and 1087 hospitalizations (Table 1).

### Male-to-female IRR

**FIG. 2.**
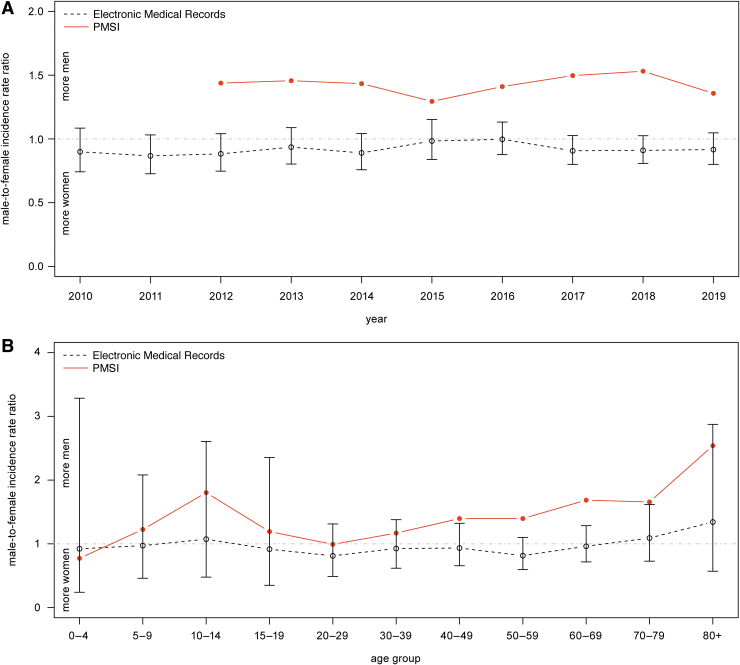
Male-to-female IRR of LB in primary care and hospital settings in France. Evolution of the male-to-female IRR in primary care (*i.e*., EMR, *gray empty circle*) and among hospitalized patients (*i.e*., PMSI, *red filled circle*) **(A)** and male-to-female IRR by age groups over 2010–2019 period in primary care and 2012–2019 period among hospitalized patients **(B)**. The lower and upper confidence intervals are provided for the EMR. IRR, incidence rate ratio.

Comparing incidence rates by sex highlighted major disparities (Fig. 2). Women were more likely to present in primary care settings compared with men (IRR = 0.92, 95% CI, 0.79–1.07) during the 10-year period (Fig. 2A). In contrast, more men were hospitalized (IRR = 1.4).

The average male-to-female IRR among hospitalized patients combining data from 2012 to 2019 revealed the higher proportion of men was predominant during adolescence (IRR for 10–14 years of age = 1.8) and gradually increased from 20 to 29 years of age until reaching a second peak at 80 years of age and older (IRR = 2.5) (Fig. 2B). In the primary care data, the average male-to-female IRR over 2010–2019 period was ∼1 for all age groups with an increase in favor of men older than 70 years of age.

### Age-specific incidence rate

**FIG. 3.**
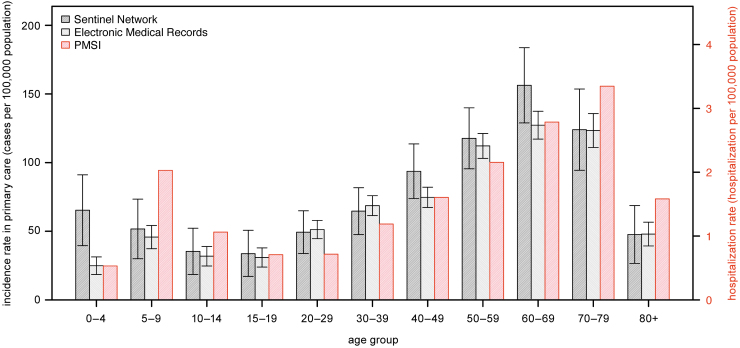
Average annual incidence rates of LB by age groups in primary care and hospital settings in France, 2017–2019. Primary care incidence rates are extrapolated from the Sentinel network (*black hashed bar*) and the EMR (*gray hashed bar*) and hospitalization rates from the PMSI (*red hashed bar*). The lower and upper confidence intervals are provided for the Sentinel network and the EMR. *Left Y-axis* represents the incidence rate in primary care and the *right Y-axis* represents the hospitalization rate.

Analysis by age for 2017–2019 highlighted a bimodal distribution of the incidence rates, with peaks in children and older adults (Fig. 3 and Table 1). In primary care, the first peak was observed in children 0–4 years of age for the Sentinel network and 5–9 years of age for the EMR. The average annual incidence rates were the lowest in adolescence (*i.e*., 10–19 years of age) for both sources, and increased gradually with age until reaching a second peak in the 60–69 age group (Sentinel network: 156.3/100,000 [95% CI, 128.9–183.7/100,000]; EMR: 127.3/100,000 [95% CI, 117.1–137.4/100,000]).

A similar age-group distribution was observed for hospitalizations, with a shift in age groups compared with primary care (Fig. 3 and Table 1): the lowest average annual incidence rate was observed in 15–29 years of age with a gradual increase with age, reaching a maximum in the 70–79 age group (3.35/100,000). Among individuals aged 14 years and younger, the incidence rate peak was observed in 5–9 years of age (2/100,000).

### Geographical variability

**FIG. 4.**
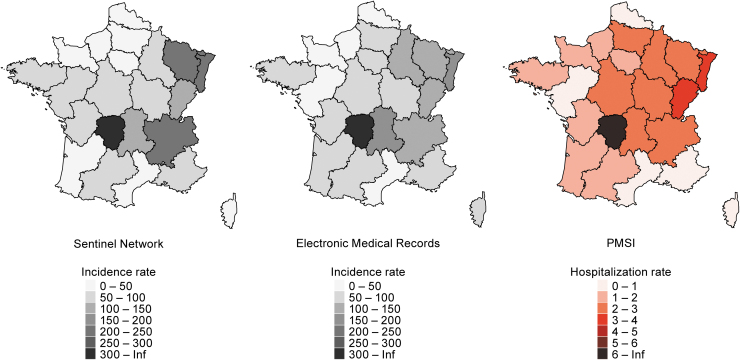
Average annual incidence rates of LB by region in primary care and hospital settings in France, 2017–2019. Primary care incidence rates are extrapolated from the Sentinel network and the EMR and hospitalization rates from the PMSI. Rates are provided in cases or hospitalization per 100,000 population per year, averaged over 2017–2019 period.

The average annual incidence rates for 2017–2019 were very different among the regions (Fig. 4 and Table 1). The incidence rates were the highest in Limousin in all three sources (Sentinel network: 330/100,000; EMR: 355.3/100,000; PMSI: 8.15/100,000), followed by Alsace (Sentinel network: 244.3/100,000; EMR: 162.3/100,000; PMSI: 3.35/100,000). Of interest, incidence rates in regions neighboring Limousin (*i.e*., Centre, Poitou-Charentes, Aquitaine, Midi-Pyrenees) were relatively low compared with Limousin. LB cases were reported in all regions over the study period, confirming LB is endemic in France, with predominance in north-eastern regions (*i.e*., Alsace, Lorraine, Champagne-Ardenne, Franche-Comté) and central-eastern regions (*i.e*., Rhône-Alpes, Auvergne, Limousin) (Fig. 4 and Table 1).

## Discussion

This study demonstrated that LB incidence in primary care almost doubled over a 10-year period. The increase observed in 2016 could have resulted from rising awareness of LB in France during this period, for example, the annual number of Google search engine requests for “Lyme” increased rapidly from 2014 to 2016 (Google, 2022). Similarly, print and TV media coverage of LB peaked in 2016 (Pascal et al, 2020). This may have increased knowledge in the general population of early LB manifestations and encouraged patients to seek medical care. During the same time period, the hospitalization rate and incidence rates of disseminated forms of LB seen in primary care, estimated by the Sentinel network, were stable (Sentinelles, 2019). However, the increased number of consultations for LB in primary care setting should have led to a proportionally decreasing number of late manifestation and associated hospitalizations, which is not observed. This should be further investigated.

This study analyzed for the first time an additional source of LB epidemiology data (*i.e*., EMR). Incidence rates from the EMR were similar to those from the Sentinel network, at national level, by region, and by age group. In addition, *ad hoc* regional prospective surveillance studies in Alsace, Franche-Comté, and Aquitaine found similar rates compared with the Sentinel network and EMR for the same periods (Sophie Raguet et al, 2018; Tessier Sabrina et al, 2018). The similarity in data from different surveillance systems suggests their capacity in estimating the epidemiology in primary care despite different specific limitations. However, similar limitations may apply to all, such as the inability to capture patients not seeking medical care.

The analyses found a slight predominance of women identified in primary care setting and more men in hospital setting. These results could not distinguish if this reflects actual differences in risks of different clinical manifestations or differences in medical care-seeking behavior. Several studies have mentioned that sex may modulate preventive behaviors, susceptibility to infection, as well as the clinical expression of LB (Aenishaenslin et al, 2015; Bennet et al, 2006; Carlsson et al, 2018; Jarefors et al, 2006; Strle et al, 2013). Of interest, the results of our study are consistent with a study performed in Slovenia where females had more cutaneous manifestations, treated in the community, whereas males had predominantly noncutaneous manifestations, treated in hospitals (Strle et al, 2013). However, not all studies have found such results (Strle et al, 2013). Comparison of the sex-specific IRR by age group between primary care and hospitalization was possible using the EMR and PMSI data and identified important differences according to age group among hospitalized patients, which were not observed in primary care. This merits further investigation.

Age-specific analysis indicated children and older adults are particularly at risk of LB. Hypotheses for this finding include higher exposure to ticks among certain age groups, absence of careful body inspection for ticks after returning from outdoor activities (Septfons et al, 2021), and increasing susceptibility to developing LB symptoms with age (Oosting et al, 2016). Of interest, hospitalization is particularly frequent in 5–9 years of age compared with primary care incidence. Explanations may include difficulty in detecting EM among children, which may lead to disseminated forms of LB. This observation should encourage parents to pay particular attention to this age group.

Limousin, located in the center of the country, had the highest incidence rate in all data sources with average incidence rates three times higher than the national average and relatively low incidence rates were observed in neighboring regions. Although Limousin is a rural region characterized by a low population density and is heavily forested, these characteristics are not unique to Limousin. According to a population survey, Limousin had the highest proportion of its population bitten by a tick, in 2016 and 2019, which may indicate a higher density of ticks in this region or a higher proportion of its population may engage in activities placing them at risk of being bitten by ticks (*e.g*., forest activities) (Septfons et al, 2021). Data from primary care and hospitalizations were consistent across regions, suggesting the criteria used to extract LB data from the PMSI database, although not disease-specific and highly dependent on coding practice, may correctly capture the relative regional trends in LB cases.

The disease surveillance systems used in this study have limitations. Estimations from the Sentinel network and the EMR are less accurate compared with regional prospective surveillance studies that include a higher number of participating GPs (7–12% of the GP population in the regional studies compared with 0.5–2% in the Sentinel Network and the EMR). Consequently, these national surveillance systems are more impacted by variation of the annual incidence, particularly when analyzing by age group or region. To mitigate the variability, the last 3 years of the study (*i.e*., 2017–2019) were pooled together.

Another limitation of the Sentinel network and the EMR is the absence of pediatricians, which may cause an underestimation in younger age groups. Pediatricians are part of the Sentinel network but do not report LB. The number of pediatricians from the EMR network is limited and is not equally distributed across regions, making extrapolation difficult. EMR data from pediatricians were not included in this study; however, an *ad hoc* analysis of the EMR confirmed notification of LB in children from participating pediatricians. In addition, without manifestation data from either of the three data sources, it is not possible to estimate the incidence of manifestations. However, GPs almost exclusively diagnose and treat EM, whereas the hospitalizations are exclusively disseminated cases, thus providing some insights into manifestation incidence.

Finally, the specificity of LB codes in the PMSI is considered low. A poor positive predictive value of Lyme code (*i.e*., 65%) was found in a study from 1999 to 2006. However, coding practices and LB awareness have improved during these years. In addition, this study was performed in one region only (*i.e*., Indre-et-Loire) and included 69 patients. Another evaluation, initiated in Limoges hospital from 2008 to 2016, highlighted the great majority of hospitalized cases recorded with Lyme disease was not owing to LB (Vidal et al, 2018). Therefore, it is possible the use of our patient selection criteria based on ICD-10 codes has led to an overestimation of hospitalization rate in this study and did not solely capture LB cases.

## Conclusions

This study provides the latest incidence rates of LB in France. Three independent sources were used for the first time, which contributed to the generation of a comprehensive epidemiological picture, including stratification by age, sex, and residence in primary care and hospital settings. Analyses highlighted specific populations at higher risk, which may help to optimize prevention programs. This study also found disparities in the sex-specific IRR and predominant age groups between primary care and hospital settings that need to be further explored. Finally, none of the sources captured patients not seeking medical care, leading to potential underestimation of LB incidence, at least LB not progressing to disseminated disease.

## Availability of Data and Materials

All data generated or analyzed during this study are included in this published article and Supplementary Tables S1 and S2. The data from PMSI that support the findings of this study are available from the Technical Agency for Information on Hospital Care—ATIH (PMSI holder). But restrictions apply to the availability of these data, which were used with an authorization for the current study, and so are not publicly available. The data from EMR that support the findings of this study are available from IQVIA, but restrictions apply to the availability of these data, which were used under license for this study and so are not publicly available. The data from Sentinel network are publicly available on their website, within the annual reports (https://www.sentiweb.fr/france/fr/?page=bilan).

## Supplementary Material

Supplemental data

Supplemental data
