# Supplementary material for: Epidemiology of Lyme Borreliosis in France in Primary Care and Hospital Settings, 2010–2019
Source: Vector Borne Zoonotic Dis. 2023 Apr 12;23(4):221–9. doi: 10.1089/vbz.2022.0050 (PMC10122229; doi:10.1089/vbz.2022.0050)
Supplement: Supplemental data [file Suppl_TableS1.docx]

Supplementary Table 1. Evolution of incidence rates of LB by region in primary care (i.e. Sentinel network, EMR) and hospital (i.e. PMSI) settings in France, 2010-2019

|  | | **Sentinel Network**  estimate (95% CI) | **EMR**  estimate (95% CI) | **PMSI** |
| --- | --- | --- | --- | --- |
| **Alsace** |  |  |  |  |
| 2010 | /100,000 | 115 (29-201) | 71.2 (41.4-100.9) | NA |
| 2011 | /100,000 | 189 (63-315) | 114.1 (77.8-150.3) | NA |
| 2012 | /100,000 | 92 (1-183) | 76.3 (45.8-106.8) | 4.73 |
| 2013 | /100,000 | 149 (34-264) | 76 (46.2-105.8) | 3.69 |
| 2014 | /100,000 | 87 (0-194) | 92.8 (58.5-127.2) | 4.75 |
| 2015 | /100,000 | 183 (66-300) | 104 (71.6-137.1) | 3.88 |
| 2016 | /100,000 | 281 (163-399) | 148 (107.4-188.7) | 3.82 |
| 2017 | /100,000 | 217 (118-316) | 107 (73.1-141.3) | 3.92 |
| 2018 | /100,000 | 265 (156-374) | 214 (166.7-260.3) | 3.38 |
| 2019 | /100,000 | 251 (127-375) | 166 (122.7-209.7) | 2.75 |
| **Aquitaine** |  |  |  |  |
| 2010 | /100,000 | 25 (0-75) | 34.2 (20.2-48.2) | NA |
| 2011 | /100,000 | 0 (0-0) | 52.8 (35.5-70) | NA |
| 2012 | /100,000 | 0 (0-0) | 70.5 (51.5-89.5) | 1.83 |
| 2013 | /100,000 | 14 (0-42) | 52.2 (35.4-69) | 1.96 |
| 2014 | /100,000 | 18 (0-52) | 56.7 (39.1-74.3) | 1.5 |
| 2015 | /100,000 | 102 (6-198) | 86 (62.7-110.1) | 1.87 |
| 2016 | /100,000 | 52 (0-127) | 107 (81-132.1) | 1.83 |
| 2017 | /100,000 | 0 (0-0) | 100 (75.3-124.6) | 2.2 |
| 2018 | /100,000 | 46 (0-94) | 90 (67-112.8) | 2.1 |
| 2019 | /100,000 | 88 (22-154) | 74 (53.4-94.4) | 1.59 |
| **Auvergne** |  |  |  |  |
| 2010 | /100,000 | 75 (18-132) | 42.9 (11.1-74.7) | NA |
| 2011 | /100,000 | 33 (0-72) | 61.6 (26.7-96.4) | NA |
| 2012 | /100,000 | 57 (11-103) | 63.3 (31.2-95.3) | 3.84 |
| 2013 | /100,000 | 44 (1-87) | 86.7 (50.5-122.9) | 4.35 |
| 2014 | /100,000 | 72 (5-139) | 126.3 (84.5-168.2) | 4.12 |
| 2015 | /100,000 | 47 (6-88) | 119 (77.2-161) | 3.16 |
| 2016 | /100,000 | 105 (41-169) | 168 (118.3-217.6) | 3.3 |
| 2017 | /100,000 | 160 (91-229) | 131 (88.3-174.1) | 2.42 |
| 2018 | /100,000 | 92 (41-143) | 185 (136.4-234.4) | 2.64 |
| 2019 | /100,000 | 92 (39-145) | 144 (101.9-186.1) | 2.64 |
| **Basse-Normandie** |  |  |  |  |
| 2010 | /100,000 | 29 (0-86) | 40.2 (5-75.4) | NA |
| 2011 | /100,000 | 40 (0-119) | 79.6 (30.2-128.9) | NA |
| 2012 | /100,000 | 149 (0-428) | 91.8 (46.8-136.9) | 1.42 |
| 2013 | /100,000 | 0 (0-0) | 48.5 (16.8-80.2) | 1.28 |
| 2014 | /100,000 | 0 (0-0) | 30.4 (3.8-57) | 1.01 |
| 2015 | /100,000 | 34 (0-72) | 21 (0-45.5) | 1.15 |
| 2016 | /100,000 | 25 (0-60) | 11 (0-31.6) | 1.56 |
| 2017 | /100,000 | 23 (0-54) | 25 (0-54.2) | 1.56 |
| 2018 | /100,000 | 12 (0-35) | 20 (0-43.5) | 0.82 |
| 2019 | /100,000 | 12 (0-35) | 25 (0.5-49.3) | 1.78 |
| **Bourgogne** |  |  |  |  |
| 2010 | /100,000 | 0 (0-0) | 58.8 (29.1-88.6) | NA |
| 2011 | /100,000 | 0 (0-0) | 60.3 (29.8-90.8) | NA |
| 2012 | /100,000 | 0 (0-0) | 87.4 (54.4-120.3) | 2.13 |
| 2013 | /100,000 | 0 (0-0) | 122 (85.1-158.8) | 2.44 |
| 2014 | /100,000 | 0 (0-0) | 66.5 (40.4-92.6) | 1.28 |
| 2015 | /100,000 | 24 (0-71) | 48 (26-70.6) | 1.77 |
| 2016 | /100,000 | 79 (0-159) | 96 (64.1-127.5) | 2.14 |
| 2017 | /100,000 | 58 (7-109) | 110 (76.5-142.7) | 3.49 |
| 2018 | /100,000 | 149 (41-257) | 117 (84-149.3) | 1.78 |
| 2019 | /100,000 | 44 (0-106) | 64 (40.2-87.4) | 1.79 |
| **Bretagne** |  |  |  |  |
| 2010 | /100,000 | 15 (0-32) | 12.8 (3.3-22.2) | NA |
| 2011 | /100,000 | 37 (0-74) | 22.7 (9.9-35.6) | NA |
| 2012 | /100,000 | 24 (0-57) | 32.2 (17.3-47.1) | 2.19 |
| 2013 | /100,000 | 65 (16-114) | 37 (21.2-52.8) | 1.9 |
| 2014 | /100,000 | 14 (0-34) | 32.9 (16.8-49) | 2.01 |
| 2015 | /100,000 | 36 (0-79) | 28 (14-42.6) | 2.16 |
| 2016 | /100,000 | 19 (0-46) | 46 (28-64.2) | 2.54 |
| 2017 | /100,000 | 22 (0-47) | 67 (45.2-88.2) | 2.05 |
| 2018 | /100,000 | 54 (12-96) | 53 (32.6-73.2) | 2.01 |
| 2019 | /100,000 | 87 (35-139) | 49 (30.1-68.8) | 1.65 |
| **Centre** |  |  |  |  |
| 2010 | /100,000 | 67 (0-146) | 23.7 (10.8-36.5) | NA |
| 2011 | /100,000 | 23 (0-55) | 46.2 (28.1-64.4) | NA |
| 2012 | /100,000 | 44 (0-90) | 35 (18.9-51.2) | 2.3 |
| 2013 | /100,000 | 32 (0-70) | 63.5 (41.5-85.5) | 2.26 |
| 2014 | /100,000 | 26 (0-63) | 33.1 (18.2-48) | 2.13 |
| 2015 | /100,000 | 30 (3-57) | 28 (14.4-42.2) | 2.25 |
| 2016 | /100,000 | 36 (5-67) | 54 (35.9-73) | 1.67 |
| 2017 | /100,000 | 47 (19-75) | 61 (40.1-81.7) | 2.02 |
| 2018 | /100,000 | 58 (26-90) | 56 (35.7-75.6) | 2.61 |
| 2019 | /100,000 | 79 (44-114) | 44 (26.9-61.7) | 3.43 |
| **Champagne-Ardenne** |  |  |  |  |
| 2010 | /100,000 | 54 (0-132) | 61.8 (35.4-88.2) | NA |
| 2011 | /100,000 | 89 (0-190) | 67.2 (40.3-94.1) | NA |
| 2012 | /100,000 | 51 (0-122) | 110.6 (71.7-149.6) | 2.91 |
| 2013 | /100,000 | 190 (21-359) | 109.7 (73.9-145.5) | 2.99 |
| 2014 | /100,000 | 68 (0-207) | 102.7 (70.9-134.6) | 2.76 |
| 2015 | /100,000 | 48 (0-106) | 77 (50-104.3) | 3.21 |
| 2016 | /100,000 | 12 (0-35) | 129 (93.5-164.2) | 2.18 |
| 2017 | /100,000 | 36 (0-77) | 125 (91.1-159) | 2.56 |
| 2018 | /100,000 | 106 (40-172) | 158 (119-197.8) | 3.71 |
| 2019 | /100,000 | 112 (41-183) | 63 (38.9-87.4) | 2.66 |
| **Corse** |  |  |  |  |
| 2010 | /100,000 | 12 (0-36) | 40.8 (0-86.9) | NA |
| 2011 | /100,000 | 39 (0-85) | 53.2 (1.1-105.3) | NA |
| 2012 | /100,000 | 35 (0-85) | 81 (16.2-145.8) | <10 cases |
| 2013 | /100,000 | 86 (0-174) | 40.1 (0-85.5) | <10 cases |
| 2014 | /100,000 | 0 (0-0) | 77 (15.4-138.6) | <10 cases |
| 2015 | /100,000 | 19 (0-46) | 85 (22.1-148.3) | <10 cases |
| 2016 | /100,000 | 32 (0-64) | 48 (1-94.6) | <10 cases |
| 2017 | /100,000 | 23 (0-49) | 83 (21.5-144.2) | <10 cases |
| 2018 | /100,000 | 19 (0-46) | 35 (0-75.4) | <10 cases |
| 2019 | /100,000 | 31 (0-62) | 63 (7.8-119.1) | <10 cases |
| **Franche-Comté** |  |  |  |  |
| 2010 | /100,000 | 25 (0-60) | 103.5 (65.1-141.8) | NA |
| 2011 | /100,000 | 148 (58-238) | 122.7 (80.2-165.2) | NA |
| 2012 | /100,000 | 66 (0-134) | 65.2 (29.8-100.7) | 3.4 |
| 2013 | /100,000 | 55 (0-120) | 183.8 (122.9-244.6) | 3.65 |
| 2014 | /100,000 | 140 (29-251) | 130 (80-180) | 2.21 |
| 2015 | /100,000 | 117 (28-206) | 88 (45-131.5) | 2.29 |
| 2016 | /100,000 | 126 (51-201) | 182 (119.7-243.7) | 1.61 |
| 2017 | /100,000 | 113 (43-183) | 117 (70.4-164.3) | 2.55 |
| 2018 | /100,000 | 111 (36-186) | 142 (89.2-194.1) | 3.57 |
| 2019 | /100,000 | 107 (27-187) | 90 (46.2-134.8) | 3.41 |
| **Haute-Normandie** |  |  |  |  |
| 2010 | /100,000 | 0 (0-0) | 12.9 (3.3-22.5) | NA |
| 2011 | /100,000 | 74 (0-183) | 23.3 (10.6-36) | NA |
| 2012 | /100,000 | 31 (0-92) | 9.6 (1.2-18.1) | 1.35 |
| 2013 | /100,000 | 22 (0-66) | 10.5 (1.3-19.7) | 1.51 |
| 2014 | /100,000 | 78 (0-166) | 18.1 (5.6-30.6) | 1.24 |
| 2015 | /100,000 | 28 (0-82) | 25 (10.8-38.9) | 1.51 |
| 2016 | /100,000 | 19 (0-55) | 24 (9.9-38.6) | 0.97 |
| 2017 | /100,000 | 66 (0-133) | 34 (16.9-51.4) | 1.56 |
| 2018 | /100,000 | 43 (0-103) | 38 (20.6-56) | 1.19 |
| 2019 | /100,000 | 27 (0-79) | 19 (7.4-31.5) | 1.24 |
| **Ile-de-France** |  |  |  |  |
| 2010 | /100,000 | 56 (27-85) | 32.6 (25.1-40.1) | NA |
| 2011 | /100,000 | 30 (6-54) | 33.9 (26.3-41.5) | NA |
| 2012 | /100,000 | 26 (3-49) | 27.4 (20.7-34.2) | 0.96 |
| 2013 | /100,000 | 37 (5-69) | 28.7 (21.9-35.6) | 1.29 |
| 2014 | /100,000 | 27 (5-49) | 36 (28.6-43.4) | 1.31 |
| 2015 | /100,000 | 38 (16-60) | 43 (34.5-51.1) | 1.17 |
| 2016 | /100,000 | 37 (8-66) | 62 (52.5-72.2) | 1.27 |
| 2017 | /100,000 | 30 (11-49) | 56 (46.8-65.6) | 1.37 |
| 2018 | /100,000 | 39 (17-61) | 57 (47.6-66.3) | 0.92 |
| 2019 | /100,000 | 44 (23-65) | 45 (37-52.9) | 1.09 |
| **Languedoc-Roussillon** | |  |  |  |
| 2010 | /100,000 | 47 (0-102) | 23.5 (7.2-39.9) | NA |
| 2011 | /100,000 | 13 (0-38) | 26.6 (9.2-44) | NA |
| 2012 | /100,000 | 0 (0-0) | 35.1 (15.2-54.9) | 0.41 |
| 2013 | /100,000 | 55 (6-104) | 20.2 (5.2-35.2) | 0.62 |
| 2014 | /100,000 | 14 (0-41) | 8.6 (0-18.3) | 0.44 |
| 2015 | /100,000 | 10 (0-30) | 8 (0-17) | <10 cases |
| 2016 | /100,000 | 22 (0-53) | 26 (9.9-42.2) | <10 cases |
| 2017 | /100,000 | 0 (0-0) | 60 (36.3-84.6) | 0.71 |
| 2018 | /100,000 | 52 (1-103) | 52 (30.5-74.4) | 1.13 |
| 2019 | /100,000 | 29 (0-62) | 30 (14.5-46.3) | 1.06 |
| **Limousin** |  |  |  |  |
| 2010 | /100,000 | 168 (0-348) | 168.5 (111.8-225.1) | NA |
| 2011 | /100,000 | 392 (124-660) | 191.6 (131.4-251.7) | NA |
| 2012 | /100,000 | 29 (0-85) | 253.7 (186-321.3) | 7.85 |
| 2013 | /100,000 | 270 (78-462) | 288 (215.1-360.9) | 8 |
| 2014 | /100,000 | 142 (17-267) | 211.5 (139.3-283.6) | 7.86 |
| 2015 | /100,000 | 516 (135-897) | 229 (154.4-304.1) | 7.59 |
| 2016 | /100,000 | 617 (318-916) | 299 (217.3-381.7) | 5.71 |
| 2017 | /100,000 | 417 (189-645) | 311 (227.3-394.8) | 8.03 |
| 2018 | /100,000 | 459 (105-813) | 297 (214-380.4) | 7.37 |
| 2019 | /100,000 | 114 (13-215) | 458 (336.7-578.7) | 9.04 |
| **Lorraine** |  |  |  |  |
| 2010 | /100,000 | 34 (0-82) | 105 (75-135) | NA |
| 2011 | /100,000 | 15 (0-44) | 95.1 (66-124.2) | NA |
| 2012 | /100,000 | 0 (0-0) | 85.9 (58.6-113.2) | 2.85 |
| 2013 | /100,000 | 48 (0-115) | 89.2 (59.7-118.8) | 3.58 |
| 2014 | /100,000 | 14 (0-41) | 49.6 (26.7-72.6) | 2.95 |
| 2015 | /100,000 | 128 (0-314) | 78 (50.3-105) | 2.73 |
| 2016 | /100,000 | 332 (172-492) | 140 (102.6-177.3) | 2.61 |
| 2017 | /100,000 | 189 (85-293) | 132 (98.6-166.1) | 2.92 |
| 2018 | /100,000 | 269 (152-386) | 107 (76.9-137.6) | 3.61 |
| 2019 | /100,000 | 165 (89-241) | 91 (61.4-120.9) | 2.33 |
| **Midi-Pyrenees** |  |  |  |  |
| 2010 | /100,000 | 50 (0-120) | 41.7 (24.3-59.1) | NA |
| 2011 | /100,000 | 14 (0-42) | 28.5 (14.1-43) | NA |
| 2012 | /100,000 | 144 (27-261) | 52.7 (33.8-71.5) | 1.4 |
| 2013 | /100,000 | 107 (19-195) | 78.5 (56.5-100.4) | 1.22 |
| 2014 | /100,000 | 70 (16-124) | 45.2 (29-61.4) | 1.54 |
| 2015 | /100,000 | 82 (19-145) | 51 (33.7-67.9) | 1.7 |
| 2016 | /100,000 | 58 (11-105) | 70 (49.5-90.4) | 1.66 |
| 2017 | /100,000 | 93 (31-155) | 108 (83.3-132.5) | 1.48 |
| 2018 | /100,000 | 96 (36-156) | 93 (70.5-115.9) | 1.38 |
| 2019 | /100,000 | 60 (22-98) | 80 (59.4-100.1) | 0.88 |
| **Nord-Pas-de-Calais** |  |  |  |  |
| 2010 | /100,000 | 0 (0-0) | 15 (6.5-23.5) | NA |
| 2011 | /100,000 | 0 (0-0) | 9.8 (3-16.5) | NA |
| 2012 | /100,000 | 0 (0-0) | 20 (10.2-29.8) | 0.44 |
| 2013 | /100,000 | 0 (0-0) | 19.2 (9.5-29) | 0.62 |
| 2014 | /100,000 | 34 (0-82) | 18 (8.6-27.4) | 0.64 |
| 2015 | /100,000 | 8 (0-24) | 17 (8-25.5) | 0.59 |
| 2016 | /100,000 | 63 (10-116) | 36 (23.4-48.9) | 0.91 |
| 2017 | /100,000 | 14 (0-34) | 34 (21.6-46.3) | 1.3 |
| 2018 | /100,000 | 62 (14-110) | 34 (21.8-46.1) | 0.81 |
| 2019 | /100,000 | 30 (0-60) | 17 (8.7-25.4) | 0.72 |
| **Pays-de-la-Loire** |  |  |  |  |
| 2010 | /100,000 | 0 (0-0) | 19.1 (10-28.2) | NA |
| 2011 | /100,000 | 0 (0-0) | 19.3 (10.1-28.5) | NA |
| 2012 | /100,000 | 27 (0-80) | 30.3 (17.9-42.6) | 0.47 |
| 2013 | /100,000 | 0 (0-0) | 27.6 (16.1-39.1) | 0.36 |
| 2014 | /100,000 | 0 (0-0) | 30.9 (19.4-42.3) | 0.79 |
| 2015 | /100,000 | 25 (0-73) | 31 (20.5-42.3) | 0.62 |
| 2016 | /100,000 | 0 (0-0) | 54 (39.7-67.6) | 0.67 |
| 2017 | /100,000 | 44 (4-84) | 47 (34.1-60.2) | 0.9 |
| 2018 | /100,000 | 34 (1-67) | 49 (35.8-61.6) | 0.8 |
| 2019 | /100,000 | 99 (48-150) | 42 (29.2-54.9) | 0.74 |
| **Picardie** |  |  |  |  |
| 2010 | /100,000 | 51 (0-123) | 36.5 (17.4-55.6) | NA |
| 2011 | /100,000 | 43 (0-126) | 52.2 (29.9-74.6) | NA |
| 2012 | /100,000 | 0 (0-0) | 19.5 (6-33) | 2.18 |
| 2013 | /100,000 | 0 (0-0) | 28.5 (13-43.9) | 2.59 |
| 2014 | /100,000 | 0 (0-0) | 30.1 (13.7-46.5) | 2.23 |
| 2015 | /100,000 | 0 (0-0) | 23 (8.9-38) | 2.48 |
| 2016 | /100,000 | 59 (0-125) | 45 (25.5-63.7) | 2.79 |
| 2017 | /100,000 | 33 (0-71) | 78 (52.5-103.4) | 2.33 |
| 2018 | /100,000 | 59 (0-118) | 61 (36.7-85.5) | 2.23 |
| 2019 | /100,000 | 21 (0-50) | 48 (27.3-68.1) | 1.56 |
| **Poitou-Charentes** |  |  |  |  |
| 2010 | /100,000 | 100 (0-213) | 37.2 (14.2-60.3) | NA |
| 2011 | /100,000 | 0 (0-0) | 40.2 (16.5-64) | NA |
| 2012 | /100,000 | 85 (0-205) | 57.8 (32.5-83.2) | 1.57 |
| 2013 | /100,000 | 103 (0-222) | 24.9 (8.6-41.2) | 1.06 |
| 2014 | /100,000 | 149 (0-302) | 60.5 (34.6-86.4) | 0.83 |
| 2015 | /100,000 | 0 (0-0) | 86 (57-114.6) | 1.05 |
| 2016 | /100,000 | 292 (82-502) | 69 (42.6-95.7) | 1.38 |
| 2017 | /100,000 | 119 (0-290) | 76 (47.9-104.3) | 1.66 |
| 2018 | /100,000 | 63 (0-151) | 52 (28.6-75.3) | 2.6 |
| 2019 | /100,000 | 0 (0-0) | 70 (44.7-94.5) | 1.61 |
| **Provence-Alpes-Cote d Azur** | |  |  |  |
| 2010 | /100,000 | 0 (0-0) | 5.2 (1-9.3) | NA |
| 2011 | /100,000 | 9 (0-27) | 11.1 (5-17.1) | NA |
| 2012 | /100,000 | 28 (0-60) | 9.5 (3.9-15.1) | 0.36 |
| 2013 | /100,000 | 0 (0-0) | 18.1 (10.4-25.9) | 0.4 |
| 2014 | /100,000 | 10 (0-29) | 21.7 (13.4-30) | 0.44 |
| 2015 | /100,000 | 6 (0-19) | 18 (10.8-25.8) | 0.38 |
| 2016 | /100,000 | 30 (0-60) | 31 (21-40.3) | 0.38 |
| 2017 | /100,000 | 31 (0-62) | 33 (23.1-43.1) | 0.48 |
| 2018 | /100,000 | 88 (38-138) | 74 (58.6-88.5) | 0.34 |
| 2019 | /100,000 | 80 (32-128) | 69 (53.7-83.8) | 0.36 |
| **Rhone-Alpes** |  |  |  |  |
| 2010 | /100,000 | 72 (41-103) | 51.1 (38.5-63.7) | NA |
| 2011 | /100,000 | 104 (69-139) | 55.4 (42.5-68.2) | NA |
| 2012 | /100,000 | 121 (82-160) | 65.6 (52-79.1) | 1.66 |
| 2013 | /100,000 | 164 (116-212) | 94.5 (78.2-110.8) | 2.03 |
| 2014 | /100,000 | 94 (55-133) | 87.7 (71.5-104) | 1.7 |
| 2015 | /100,000 | 65 (37-93) | 59 (45.9-71.8) | 1.67 |
| 2016 | /100,000 | 156 (114-198) | 98 (81.6-114.5) | 1.71 |
| 2017 | /100,000 | 167 (125-209) | 114 (96.5-132.4) | 2.2 |
| 2018 | /100,000 | 315 (255-375) | 128 (109.3-147.5) | 1.81 |
| 2019 | /100,000 | 137 (98-176) | 92 (76.4-108.5) | 2.03 |
| LB: Lyme borreliosis; EMR: Electronic Medical Records; PMSI: French national hospital discharge database. | | | | |
