# Supplementary material for: Epidemiology of Lyme Borreliosis in France in Primary Care and Hospital Settings, 2010–2019
Source: Vector Borne Zoonotic Dis. 2023 Apr 12;23(4):221–9. doi: 10.1089/vbz.2022.0050 (PMC10122229; doi:10.1089/vbz.2022.0050)
Supplement: Supplemental data [file Suppl_TableS2.docx]

Supplementary Table 2. Evolution of incidence rates of LB by age group in primary care (i.e. Sentinel network, EMR) and hospital (i.e. PMSI) settings in France, 2010-2019

|  |  | **Sentinel Network**  estimate (95% CI) | **EMR**  estimate (95% CI) | **PMSI** |
| --- | --- | --- | --- | --- |
| **0-4 years** |  |  |  |  |
| 2010 | /100,000 | 18 (0-48) | 15.1 (5.2-25) | NA |
| 2011 | /100,000 | 14 (0-35) | 15.7 (7.1-24.3) | NA |
| 2012 | /100,000 | 8 (0-19) | 24.9 (13.1-36.7) | 0.57 |
| 2013 | /100,000 | 9 (0-27) | 18 (8.5-27.6) | 0.8 |
| 2014 | /100,000 | 36 (0-75) | 12.2 (4.6-19.9) | 0.86 |
| 2015 | /100,000 | 11 (0-38) | 17.2 (7.8-26.7) | 0.6 |
| 2016 | /100,000 | 12 (0-31) | 21.9 (11.4-32.4) | 0.74 |
| 2017 | /100,000 | 57 (12-102) | 22.2 (11.8-32.7) | 0.54 |
| 2018 | /100,000 | 68 (23-113) | 28 (16.2-39.8) | 0.41 |
| 2019 | /100,000 | 71 (27-115) | 24.5 (13.3-35.7) | 0.64 |
| **5-9 years** |  |  |  |  |
| 2010 | /100,000 | 50 (1-99) | 16.9 (7.4-26.3) | NA |
| 2011 | /100,000 | 27 (0-56) | 27.4 (15.6-39.2) | NA |
| 2012 | /100,000 | 29 (0-67) | 32.2 (19.7-44.6) | 2.41 |
| 2013 | /100,000 | 0 (0-0) | 28.7 (17.1-40.3) | 2.55 |
| 2014 | /100,000 | 23 (0-58) | 37.1 (23.4-50.7) | 2.04 |
| 2015 | /100,000 | 33 (0-74) | 23.7 (13-34.5) | 1.93 |
| 2016 | /100,000 | 55 (9-101) | 34.7 (22.1-47.2) | 1.52 |
| 2017 | /100,000 | 60 (17-103) | 50.3 (35.1-65.5) | 2.05 |
| 2018 | /100,000 | 60 (20-100) | 45.6 (30.8-60.4) | 2.14 |
| 2019 | /100,000 | 35 (7-63) | 41.4 (27.5-55.3) | 1.9 |
| **10-14 years** |  |  |  |  |
| 2010 | /100,000 | 17 (0-52) | 26.6 (15.1-38.1) | NA |
| 2011 | /100,000 | 9 (0-28) | 28.1 (15.9-40.2) | NA |
| 2012 | /100,000 | 0 (0-0) | 27.7 (15.2-40.2) | 1.62 |
| 2013 | /100,000 | 34 (0-74) | 30 (17.5-42.5) | 1.32 |
| 2014 | /100,000 | 5 (0-15) | 23.6 (12.6-34.6) | 1.16 |
| 2015 | /100,000 | 40 (2-78) | 31.3 (19.1-43.6) | 0.98 |
| 2016 | /100,000 | 43 (2-84) | 55.5 (38.2-72.9) | 1.16 |
| 2017 | /100,000 | 23 (0-46) | 36.7 (23.4-49.9) | 1.18 |
| 2018 | /100,000 | 51 (15-87) | 28.2 (16.8-39.7) | 0.98 |
| 2019 | /100,000 | 32 (5-59) | 30.6 (18.4-42.8) | 1.03 |
| **15-19 years** |  |  |  |  |
| 2010 | /100,000 | 9 (0-28) | 15.1 (6.5-23.8) | NA |
| 2011 | /100,000 | 22 (0-45) | 15.8 (6.8-24.9) | NA |
| 2012 | /100,000 | 0 (0-0) | 20 (9.7-30.4) | 0.58 |
| 2013 | /100,000 | 34 (1-67) | 22.8 (11.8-33.7) | 0.93 |
| 2014 | /100,000 | 18 (0-45) | 24.2 (13.5-35) | 0.87 |
| 2015 | /100,000 | 23 (0-54) | 24.3 (13.3-35.4) | 0.88 |
| 2016 | /100,000 | 57 (1-113) | 26.7 (15.4-38) | 0.95 |
| 2017 | /100,000 | 22 (0-47) | 36.3 (23.1-49.6) | 0.71 |
| 2018 | /100,000 | 31 (2-60) | 30.5 (18.8-42.2) | 0.5 |
| 2019 | /100,000 | 48 (14-82) | 25.9 (14.7-37.1) | 0.91 |
| **20-29 years** |  |  |  |  |
| 2010 | /100,000 | 40 (13-67) | 36.4 (26.4-46.4) | NA |
| 2011 | /100,000 | 39 (10-68) | 31.5 (21.9-41) | NA |
| 2012 | /100,000 | 32 (7-57) | 31.2 (22.3-40.2) | 0.53 |
| 2013 | /100,000 | 22 (0-48) | 41.3 (31-51.6) | 0.8 |
| 2014 | /100,000 | 7 (0-21) | 32.8 (23.7-41.9) | 0.56 |
| 2015 | /100,000 | 4 (0-10) | 35.1 (25.5-44.7) | 0.61 |
| 2016 | /100,000 | 43 (13-73) | 39.7 (29.8-49.7) | 0.84 |
| 2017 | /100,000 | 30 (10-50) | 46.3 (35.3-57.2) | 0.76 |
| 2018 | /100,000 | 80 (44-116) | 57.8 (45.7-70) | 0.8 |
| 2019 | /100,000 | 38 (16-60) | 49.6 (38.2-61) | 0.59 |
| **30-39 years** |  |  |  |  |
| 2010 | /100,000 | 41 (15-67) | 30.8 (22-39.7) | NA |
| 2011 | /100,000 | 40 (13-67) | 46.2 (35.3-57) | NA |
| 2012 | /100,000 | 84 (37-131) | 39.4 (29.4-49.4) | 0.96 |
| 2013 | /100,000 | 51 (18-84) | 48.9 (37.9-59.8) | 1.17 |
| 2014 | /100,000 | 68 (26-110) | 45.2 (34.8-55.7) | 1.23 |
| 2015 | /100,000 | 55 (17-93) | 46.6 (36-57.2) | 1.03 |
| 2016 | /100,000 | 86 (45-127) | 61.1 (49.1-73.1) | 1.07 |
| 2017 | /100,000 | 44 (19-69) | 61.7 (49.8-73.6) | 1.45 |
| 2018 | /100,000 | 79 (45-113) | 78.2 (64.8-91.6) | 1.13 |
| 2019 | /100,000 | 71 (42-100) | 66.1 (53.8-78.4) | 0.98 |
| **40-49 years** |  |  |  |  |
| 2010 | /100,000 | 43 (15-71) | 41.1 (31.2-50.9) | NA |
| 2011 | /100,000 | 39 (14-64) | 39.9 (30.5-49.3) | NA |
| 2012 | /100,000 | 18 (0-36) | 58.8 (47.3-70.4) | 1.63 |
| 2013 | /100,000 | 44 (15-73) | 56 (44.7-67.2) | 1.52 |
| 2014 | /100,000 | 24 (2-46) | 52.7 (41.8-63.5) | 1.48 |
| 2015 | /100,000 | 65 (28-102) | 46.9 (36.9-56.9) | 1.58 |
| 2016 | /100,000 | 83 (46-120) | 100.3 (85.4-115.1) | 1.33 |
| 2017 | /100,000 | 78 (47-109) | 94.6 (80.3-108.9) | 1.78 |
| 2018 | /100,000 | 111 (73-149) | 78.8 (65.7-91.9) | 1.76 |
| 2019 | /100,000 | 92 (58-126) | 50.8 (40.1-61.4) | 1.28 |
| **50-59 years** |  |  |  |  |
| 2010 | /100,000 | 56 (23-89) | 52.3 (40.9-63.7) | NA |
| 2011 | /100,000 | 77 (40-114) | 69.4 (56.1-82.6) | NA |
| 2012 | /100,000 | 40 (11-69) | 65.2 (52.8-77.6) | 2.12 |
| 2013 | /100,000 | 124 (75-173) | 80 (66.4-93.7) | 2.32 |
| 2014 | /100,000 | 58 (24-92) | 66.5 (54.1-78.9) | 1.91 |
| 2015 | /100,000 | 74 (27-121) | 65.3 (53.1-77.4) | 1.85 |
| 2016 | /100,000 | 83 (46-120) | 104.6 (89.2-120) | 2.25 |
| 2017 | /100,000 | 80 (48-112) | 111.1 (95.4-126.8) | 2.25 |
| 2018 | /100,000 | 166 (120-212) | 129.7 (113-146.5) | 2.18 |
| 2019 | /100,000 | 107 (71-143) | 95.7 (81.1-110.3) | 2.04 |
| **60-69 years** |  |  |  |  |
| 2010 | /100,000 | 79 (31-127) | 60.4 (46.7-74.1) | NA |
| 2011 | /100,000 | 98 (51-145) | 82.3 (66.1-98.6) | NA |
| 2012 | /100,000 | 129 (55-203) | 85.1 (69.5-100.8) | 2.85 |
| 2013 | /100,000 | 129 (78-180) | 97 (81-113) | 2.92 |
| 2014 | /100,000 | 103 (55-151) | 96.8 (80.9-112.6) | 2.74 |
| 2015 | /100,000 | 103 (51-155) | 92.4 (76.8-107.9) | 2.69 |
| 2016 | /100,000 | 198 (135-261) | 126.6 (109-144.2) | 2.56 |
| 2017 | /100,000 | 140 (92-188) | 137.8 (119.4-156.3) | 2.98 |
| 2018 | /100,000 | 184 (133-235) | 136.1 (118.2-154) | 2.56 |
| 2019 | /100,000 | 145 (102-188) | 107.8 (91.4-124.2) | 2.81 |
| **70-79 years** |  |  |  |  |
| 2010 | /100,000 | 26 (0-53) | 57.9 (41.7-74.1) | NA |
| 2011 | /100,000 | 24 (0-51) | 63.8 (46.5-81.2) | NA |
| 2012 | /100,000 | 46 (0-97) | 62.3 (46.2-78.5) | 2.29 |
| 2013 | /100,000 | 69 (16-122) | 78.7 (60.6-96.8) | 3.02 |
| 2014 | /100,000 | 35 (1-69) | 66.6 (49.5-83.7) | 3.07 |
| 2015 | /100,000 | 82 (18-146) | 73.8 (56.3-91.3) | 3.31 |
| 2016 | /100,000 | 149 (78-220) | 110.2 (88.8-131.6) | 2.99 |
| 2017 | /100,000 | 128 (75-181) | 121.3 (99.4-143.2) | 3.59 |
| 2018 | /100,000 | 149 (92-206) | 142.9 (120-165.9) | 3.35 |
| 2019 | /100,000 | 95 (52-138) | 105.9 (86.5-125.2) | 3.1 |
| **80+ years** |  |  |  |  |
| 2010 | /100,000 | 49 (0-100) | 23.2 (11.3-35) | NA |
| 2011 | /100,000 | 0 (0-0) | 14.8 (5.5-24.1) | NA |
| 2012 | /100,000 | 33 (0-72) | 17.4 (7.2-27.7) | 1.98 |
| 2013 | /100,000 | 0 (0-0) | 27.9 (15.8-40.1) | 1.49 |
| 2014 | /100,000 | 25 (0-65) | 25.4 (14.2-36.7) | 1.59 |
| 2015 | /100,000 | 25 (0-54) | 25.4 (14.4-36.4) | 1.69 |
| 2016 | /100,000 | 34 (0-70) | 37.8 (24.1-51.4) | 1.4 |
| 2017 | /100,000 | 47 (13-81) | 33.7 (21.1-46.3) | 1.73 |
| 2018 | /100,000 | 63 (19-107) | 47 (32.3-61.7) | 1.53 |
| 2019 | /100,000 | 33 (3-63) | 63.2 (45.8-80.5) | 1.49 |
| LB: Lyme borreliosis; EMR: Electronic Medical Records; PMSI: French national hospital discharge database. | | | | |
